# Psychosocial benefits of workplace physical exercise: cluster randomized controlled trial

**DOI:** 10.1186/s12889-017-4728-3

**Published:** 2017-10-10

**Authors:** Markus D. Jakobsen, Emil Sundstrup, Mikkel Brandt, Lars L. Andersen

**Affiliations:** 1 National Research Centre for the Working Environment, Lersø Parkalle 105, 2100 Copenhagen, Denmark; 2 Department of Health Science and Technology, Physical Activity and Human Performance group, SMI, Aalborg University, Aalborg, Denmark

**Keywords:** Vitality, Mental health, Pain, Biopsychosocial, Musculoskeletal disorders, Occupational health, Strength training, Patient handling, Social capital

## Abstract

**Background:**

While benefits of workplace physical exercise on physical health is well known, little is known about the psychosocial effects of such initiatives. This study evaluates the effect of workplace versus home-based physical exercise on psychosocial factors among healthcare workers.

**Methods:**

A total of 200 female healthcare workers (Age: 42.0, BMI: 24.1) from 18 departments at three hospitals were cluster-randomized to 10 weeks of: 1) home-based physical exercise (HOME) performed alone during leisure time for 10 min 5 days per week or 2) workplace physical exercise (WORK) performed in groups during working hours for 10 min 5 days per week and up to 5 group-based coaching sessions on motivation for regular physical exercise. Vitality and mental health (SF-36, scale 0–100), psychosocial work environment (COPSOQ, scale 0–100), work- and leisure disability (DASH, 0–100), control- (Bournemouth, scale 0–10) and concern about pain (Pain Catastrophizing Scale, scale 0–10) were assessed at baseline and at 10-week follow-up.

**Results:**

Vitality as well as control and concern about pain improved more following WORK than HOME (all *p* < 0.05) in spite of increased work pace (*p* < 0.05). Work- and leisure disability, emotional demands, influence at work, sense of community, social support and mental health remained unchanged. Between-group differences at follow-up (WORK vs. HOME) were 7 [95% confidence interval (95% CI) 3 to 10] for vitality, −0.8 [95% CI -1.3 to −0.3] for control of pain and −0.9 [95% CI -1.4 to −0.5] for concern about pain, respectively.

**Conclusions:**

Performing physical exercise together with colleagues during working hours was more effective than home-based exercise in improving vitality and concern and control of pain among healthcare workers. These benefits occurred in spite of increased work pace.

**Trial registration:**

NCT01921764 at ClinicalTrials.gov. Registered 10 August 2013.

## Background

Musculoskeletal pain is the primary cause of sickness absence, lost productivity and early retirement across Europe and the United States [1–6]. Although, work-related development of pain is associated with physical factors such as strenuous labor and manual handling activities [7–9] other non-biological mechanisms can influence pain perception. The biopsychosocial model of pain suggests that pain perception is a product of a multifactorial interaction between biological, psychological and social factors [10]. In view of this, optimal strategies for prevention and rehabilitation of pain should focus not only on the physiological factors, e.g. reducing workload or performing physical exercise as a single element, but rather aim at incorporating all three elements, physiological, psychological and social, in a multidisciplinary intervention.

As time spent at work comprises a large fraction of our day it is inevitable that our work will have an impact on our physical- as well as social- and psychological wellbeing. In view of the biopsychosocial model of pain, the workplace, therefore, represents an important and optimal setting for relieving pain and promoting overall employee health. Indeed systematic reviews have shown that workplace based physical exercise, especially strength training, performed together with colleagues is effective in preventing and rehabilitating musculoskeletal pain and improving physical capacity [11–14]. Although these interventions primarily focus on reducing pain they may potentially incorporate and target each element of the bio-psychosocial model. However, little is known about the psychosocial effects of such workplace initiatives. A recent review of workplace interventions on mental health disorders, nevertheless, indicate that workplace based physical exercise may reduce anxiety and depression symptoms among workers suffering from these problems, however, research is needed regarding exercise intensity and frequency of the sessions [15]. Among slaughterhouse workers with chronic pain, group-based strength training at the workplace improved vitality and social climate [16]. Among healthcare workers, physical exercise at the workplace improved working relationships within teams but not between teams and management [17]. Yet another study found no effect of a multifaceted intervention including physical exercise on support from management [18]. Thus, research investigating whether physical exercise at the workplace can lead to improvements in the psychosocial working environment and other psychosocial factors is needed.

Willingness from management to implement workplace initiatives is often dependent on the cost, i.e. money spent on working hours, instructors and training equipment. A potential cost-effective alternative to maintain employee health may be to provide the employees with training equipment and guidelines and encourage them to perform physical exercise during leisure time. On the other hand, supervised and group-based interventions (i.e. at the department) seem to enhance exercise adherence compared with home-based exercise interventions [19, 20]. Moreover, as exercising together with colleagues may improve social relations compared with exercising alone [17], it may be hypothesized that exercising alone does not promote psychosocial benefits to the same extent as exercising together with colleagues.

This article presents a secondary analysis that evaluates the effect of workplace versus home-based physical exercise on psychosocial factors among healthcare workers.

## Methods

### Study design

The study protocol and primary outcome (change in average muscle pain intensity of the low back, neck and shoulder) of this trial has previously been published elsewhere [21, 22]. The data presented in this article represents a secondary analysis of this trial.

Briefly, we conducted a two-armed parallel-group, single-blind, cluster randomized controlled trial with allocation concealment among eighteen departments from three hospitals situated in Copenhagen, Denmark. The study was performed from August 2013 to January 2014. Participants were randomly assigned to a 10-week intervention of either physical exercise performed at the workplace or at home.

### Recruitment and randomization of participants

**Fig. 1:**
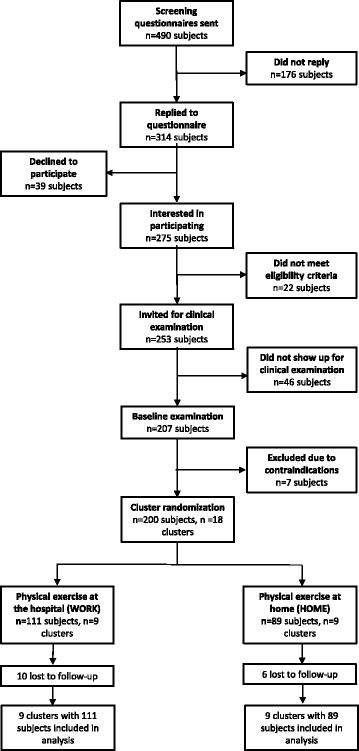
Flow of participants throughout the study

The recruitment was 2-phased and consisted of a short screening questionnaire conducted in June 2013, followed by a baseline clinical examination and questionnaire performed in Aug-Sept 2013. Figure 1 shows the overall flow of participants through the trial. Two-hundred and fifty three healthy healthcare workers, out of the 490 healthcare workers who received the screening questionnaire, were invited for a clinical examination in August and September 2013. A total of 207 employees participated in the baseline clinical examination.

Eighteen departments, with 200 participants, were randomized, using a computer-generated random numbers table, to receive either physical exercise at home (HOME) or at the workplace (WORK). All examiners were blinded to the group allocation at follow-up testing (i.e. post intervention in Dec 2013-Jan 2014). Table 1 present baseline characteristics of all participants.

**Table 1:** Characteristics of study participants (HOME and WORK). Values are reported as Mean (SD)

	WORK		HOME	
N	111		89	
Age (years)	40*	(12)	44	(10)
Height (cm)	168.4	(6.2)	168.0	(7.2)
Weight (kg)	67.5	(12.1)	68.9	(12.2)
BMI (kg∙m^−2^)	23.8	(3.8)	24.4	(4.0)
Average pain intensity in the back, neck and shoulders (0–10)	2.9	(2.1)	3.2	(2.3)
Weekly working hours	35	(4)	34	(4)
Seniority	15	(11)	18	(11)
Mental health (0–100)	81	(13)	81	(12)
Vitality (0–100)	64	(20)	66	(19)
Control of pain (0–10)	2.7	(2.5)	2.7	(2.4)
Concern about pain (0–10)	2.5	(2.7)	2.5	(2.7)
Work disability (0–100)	8.8	(15.9)	10.5	(15.8)
Leisure disability (0–100)	11.3	(15.4)	10.7	(18.7)
Emotional demands (0–100)	46	(19)	46	(18)
Influence at work (0–100)	38	(20)	39	(20)
Work pace (0–100)	69	(17)	71	(17)
Sense of community (0–100)	15	(19)	15	(15)
Social support (0–100)	24	(24)	18	(21)

HOME: Home-based physical exercise, WORK: Work-based physical exercise

* difference between groups at baseline, *P* < 0.05

### Interventions

The interventions have previously been described in detail elsewhere [21]. In brief, participants in each cluster were allocated to a 10-week intervention period receiving either 10 min of physical exercise 5 days per week at home or at the hospital. Participants randomized to workplace physical exercise (WORK, *n* = 111 subjects, *n* = 9 clusters) performed group-based and supervised strength training, during working hours at the hospital, using elastic bands (TheraBand®), kettlebells and swiss balls (Duraball Pro®). All training sessions took place in designated rooms located at or close to the respective departments and were supervised by experienced training instructors who ensured training progression. The training sessions were performed as a circuit training program which consisted of 4–6 exercises of the flowing 10 exercises: lateral raises, lunges, squeeze, golf swings and woodchoppers using elastic tubing, kettlebell deadlifts, kettlebell swings and abdominal crunches, back extensions and squats using a swissball. The instructors aimed at an intensity of 12 repetition maximum (RM) for every exercise. The participants in WORK were also offered 5 group-based motivational coaching sessions (30–45 min. With 5–12 participants in each session) during working hours. The content of the coaching sessions has been described in detail in the study protocol [21].

Participants randomized to home-based physical exercise (HOME, *n* = 89 participants, *n* = 9 clusters) performed physical exercise at home during leisure. After the participants were informed about group allocation they received a bag with elastic tubing (easy, medium, and hard elastic tubing) and 3 posters that visually demonstrated the exercises that should be performed for the shoulder-, back- and abdominal muscles [23–25]. The participants were instructed to exercise for 10 min, 5 days per week using at least 4 exercises per session of the 10 different exercises shown in the 3 posters.

### Outcome variables

The participants replied to a questionnaire concerning psychosocial factors at baseline and again at 10-week follow-up. Mental health was determined using 4 questions from the 36-item Short Form Health Survey (SF-36) [26]. The questions were “How much of the time during the past 4 weeks....” have you been a very nervous person?, have you felt so down in the dumps that nothing could cheer you up?, have you felt calm and peaceful?, have you felt downhearted and blue?. The participants replied on a 6-point scale from “All the time” to “None of the time”, and the responses were converted to a score of 0 to 100 (higher score is better).

Vitality was determined using 3 questions based on SF-36 [26]. The questions were: “How much of the time during the past 4 weeks....” did you feel full of pep?, did you have a lot of energy?, did you feel worn out?. The participants responded on a 6-point scale from “All the time” to “None of the time” which was converted to a score of 0 to 100 (higher score is better).

Psychosocial working conditions were determined using five questions from the second version of the Copenhagen Psychosocial Questionnaire (COPSOC) [27]. The participants were asked “How often…” does your work put you in emotionally disturbing situations?, do you have a large degree of influence concerning your work?, do you have to work very fast? do you feel part of a community at your place of work? How often is your immediate superior willing to listen to your work related problems?. Participants replied on a 5-point scale from “Always” to “Never”. The answers were converted to a score of 0 to 100 (higher score is worse).

Participants rated work disability at baseline and follow-up using a modified version of the work module of the Disability of the Arm Shoulder and Hand (DASH) questionnaire. Instead of only focusing on disability of the arm shoulder and hand the questions were directed to pain in general: “Did you, because of your pain within the last week, have any difficulty in...” doing your usual work?, using your usual technique for your work?, doing your work as well as you would like?, working for extended periods of time? Participants replied on a 5-point from “No difficulty” to “Unable”. The score was normalized on a scale of 0 to 100, where 100 represents the highest level of disability [28]. Participants also rated leisure time disability at baseline and follow-up using a modified version of the work module of the Disability of the Arm Shoulder and Hand (DASH) questionnaire [28]: “Did you, because of your pain within the last week, have any difficulty in...” focusing? exercising or doing sports? doing normal chores at home? Participants replied on the same 5-point from “No difficulty” to “Unable” and the score was converted in to a scale of 0 to 100, where 100 represents the highest level of disability.

The participants also rated their control of pain, on a 0 to 10 scale (10 is no control), using a single question from The Bournemouth Questionnaire [8]: “How much have you been able to control (reduce/help) your back pain on your own?”. Concern about pain was furthermore rated, on a 0 to 10 scale (10 is all the time), using a single question from The Pain Catastrophizing Scale Questionnaire [29]: “How much do you worry about whether the pain will end”.

### Statistical analysis

The statistical analyses used in the present study were performed using the SAS statistical software version 9.4 for Windows (SAS Institute, Cary, NC). Vitality, mental health, disability, psychosocial work environment, control and concern about pain was evaluated using a repeated-measures linear mixed model (Proc Mixed) with *group*, *time* and *group by time* as independent variables. Participants nested within department was entered as random effect. The statistical analyses were performed in accordance with the intention-to-treat principle, i.e. using the mixed procedure which accounts for missing values (under the assumption that they are missing at random). All analyses were adjusted for age and the respective baseline value of the outcome measure. Outcomes are reported as between-group differences and 95% confidence intervals at follow-up. An alpha level of 0.05 was accepted as statistically significant.

## Results

Table 1 shows baseline characteristics; demographics, vitality, mental health, disability, psychosocial work environment, concern and control of pain for the two intervention groups. At baseline, participants randomized to HOME were older than WORK (*p* < 0.05). Accordingly, all analyses were adjusted for age. Training adherence differed between the groups (*p* < 0.001). WORK trained on average 2.2 (SD: 1.1), whereas HOME trained 1.0 (SD: 1.2) training sessions per week. The participants in WORK attended, on average, 2.1 coaching sessions of the 5 offered coaching sessions.

Table 2 shows within-group changes and between-group differences at follow-up in vitality, mental health, disability, psychosocial work environment, control and concern about pain. G*roup by time* interactions were observed for vitality, control and concern about pain (*p* < 0.05) which corresponded to small effect sizes (Cohen d = 0.27–0.36) in favor of workplace-based physical exercise. Work pace, however, increased more in WORK compared with HOME (p < 0.05). Mental health, work disability, emotional demands, influence at work, sense of community and social support from managers remained unaltered. Finally, a tendency for a main effect (*p* = 0.08) was seen for leisure time disability.

**Table 2:** Changes in vitality, mental health, disability, psychosocial work environment, control and concern of pain from baseline to 10-week follow-up. Differences of each group are shown in left columns, while contrasts between the groups are listed in right columns. Values are means (95% confidence interval)

	Within-group difference from baseline to follow-up				Between-group difference at follow-up			
	WORK		HOME		WORK VS HOME			
	Mean	95% CI	Mean	95% CI	Mean	95% CI	*P*	Effect size
Mental health (0–100)	0.8	(−1.5–3.1)	0.2	(−2.3–2.7)	0.7	(−1.7–3.2)	0.5633	0.06
Vitality (0–100)	5	(2–8)	-2	(−3.7–3.1)	7	(3–10)	0.0003*	0.36
Control of pain (0–10)	−0.3	(−0.8–0.1)	0.4	(−0.2–0.9)	−0.8	(−1.3 - -0.3)	0.0035*	0.33
Concern about pain (0–10)	−0.6	(−1 - -0.2)	0.3	(−0.1–0.8)	−0.9	(−1.4 - -0.5)	<.0001*	0.33
Work disability (0–100)	−1.6	(−4.6–1.4)	0.7	(−2.7–4)	−3.1	(−6.4–0.2)	0.0635	0.20
Leisure disability (0–100)	−2.8	(−5.8–0.2)	1.1	(−2.2–4.4)	−3.9	(−7.2 - -0.7)	0.0171(*)	0.23
Emotional demands (0–100)	3	(−0.6–6.5)	1.4	(−2.4–5.3)	1.6	(−2.2–5.5)	0.4054	0.09
Influence at work (0–100)	−0.5	(−3.7–2.8)	−1.8	(−5.2–1.7)	1	(−2.4–4.5)	0.5588	0.05
Work pace (0–100)	5.1	(2.2–7.9)	−0.10	(−3.2–3.1)	4.6	(1.5–7.7)	0.0042*	0.27
Sense of community (0–100)	−2.2	(−5.1–0.8)	−0.8	(−4–2.5)	−1.2	(−4.4–1.9)	0.4432	0.07
Social support (0–100)	2.5	(−1.6–6.5)	3.50	(−0.9–8)	−0.4	(−4.8–4)	0.871	0.02

HOME: Home-based physical exercise, WORK: Work-based physical exercise

* denotes significant group-by-time interaction

The within-group changes from baseline to follow-up in control and concern about pain and leisure disability were significantly related to the change in average pain intensity of the neck, shoulder and lower back (Spearman rho = 0.26, *p* < 0.001; rho = 0.33, p < 0.001; rho = 0.31, p < 0.001, respectively). The changes in mental health, vitality, work disability, emotional demands, influence at work, work pace, sense of community and social support from managers, however, were unrelated to the change in average pain intensity (rho < 0.15, *p* > 0.05).

## Discussion

The present study demonstrates that workplace-based physical exercise is more effective than home-based exercise in improving vitality and concern and control of pain among healthcare workers. Thus, performing physical exercise together with colleagues at the workplace seems to induce some psychosocial benefits compared with exercising at home.

Compared with exercising at home, the average vitality score improved seven points (0–100 scale) following supervised group-based physical exercise at work. Accordingly, the healthcare workers who performed physical exercise together with colleagues were less worn out and more energetic after the ten weeks. Interestingly, the change in vitality was unrelated to pain reduction. Accordingly, other mechanisms than improvements in pain caused the change in vitality in WORK. An apparent difference between the two interventions is the fact that the workplace group exercised in groups with colleagues during working hours whereas the home group performed the exercises alone during leisure time. As exercising at work has shown to improve psychological factors such as mood and enthusiasm [30, 31], performing exercises with colleagues during working hours may not only increase physical capacity, but also increase energy level and mood through “fun” non work-related active breaks. In support of this, we have previously shown that exercising in groups at work reduces physical exertion during work and need for recovery compared to exercising alone at home among healthcare workers [32]. Thus, the improvement of vitality may also be related to the fact that the healthcare workers improved their physical capacity, i.e. leading to a higher reserve capacity and thus higher levels of vitality. On the other hand, another recent workplace study was unable to demonstrate changes in vitality after one year of yoga and other exercise activities performed during working hours among hospital workers [33]. The authors, however, argued that the lack of changes was a result of poor implementation and a potential ceiling effect due to the workers’ relative high health scores that were close to the upper limit. A recent study by Matsugaki et al. found that supervised physical exercise among nurses improved depressive symptoms compared to exercising without supervision [34]. Thus, the provision of supervised instruction may potentially explain some of the improvements found following the 10 weeks of supervised group-based exercise at work. Yet, a study conducted among nursing home employees did not find improvements in health-related quality of life following 6 months of supervised physical exercise [35].

Mental health did not change in this study. As argued above, this may be due to a ceiling effect caused by the relatively high mental health scores at baseline (81 on a 0–100 scale) which leaves little room for improvements. This is in contrast with previous studies demonstrating improvements in mental health in response to exercise. However, these studies were conducted among workers with relative poor mental health scores [15] and patients suffering from i.e. depression and anxiety [36]. Accordingly, performing exercise may improve mental health among workers with poor mental health, but may not provide additional benefits to mentally healthy adults.

Concern about pain and control of pain improved more following workplace-based exercise compared with exercising alone. The change scores in concern and control of pain were, not surprisingly, related to the reduction in average pain intensity in the neck, shoulder and lower back (rho = 0.26–0.33). Accordingly, by relieving pain through workplace-based exercise the healthcare workers gained more control of their pain as well as became less worried about whether the pain would end. Thus, it seems that exercise at the workplace may evoke a positive cycle where an initial reduction in pain may improve the workers belief that pain is modifiable and therefore motivate the workers to continue with the exercises. However, the impact of the instructor and the coaching sessions should not be neglected as they incorporated ways to maintain motivation for doing the exercises. Thus, the context of the present study – i.e. physical exercise at work with support from coaches – may be the key to achieve the observed benefits.

The improvements in pain intensity and control and concern about pain in WORK did, however, not affect the between-group difference in work disability at follow-up. In support of this, the reduction in pain intensity was unrelated to the changes in work disability, i.e. changes in how the workers were using their usual technique, how well they could perform the job as well as they liked and whether they could work for extended periods. Notably, a tendency for a *group by time* effect (*p* = 0.08) was observed for leisure disability where the within-group change was related to pain relief (rho = 0.31). Thus, reduction in musculoskeletal pain seems to have a larger impact on disability during leisure, i.e. difficulty in focusing or doing sports and chores at home rather than work disability. This is somewhat in contrast with our previous findings among slaughterhouse workers with chronic pain and high disability in the arm, shoulder and hand (>28 on 0–100 scale) who altered work disability following 10 weeks of workplace exercise [37]. Nevertheless, the present results may have been influenced by a floor effect as both work and leisure disability were relatively low (approximately 10 on a 0–100 scale, where 0 is no disability). On the other hand, studies conducted among metal workers and female workers with disability scores comparable to the present study, have recently provided evidence that tailored workplace exercise programs are effective in improving disability of the upper limbs [38, 39]. Hence, a more personalized approach focusing on the individual needs may be more effective than a general exercise program. Yet, the former approach increases the need for individual instructions and exercises which may compromise the effect of collectively exercising together. Individual instructions and handling of individual data may, moreover, lead to further expenses in terms of instructor employment and working hours spent.

We also measured psychosocial work environment using five items from the COPSOQ-questionnaire. Interestingly, despite exercising together with colleagues, the workplace group did not improve sense of community at the work place compared with the home group. Similarly, a recent study by Chanchai and co-workers was unable to demonstrate changes in sense of community in response to a participatory ergonomic intervention aiming at reducing musculoskeletal disorders and psychosocial risk factors among hospital orderlies [40]. By contrast, in a previous analysis from the present randomized controlled trial, we have shown that performing group-based physical exercise at the workplace improves social capital within working teams among healthcare workers [17]. This controversy may be related to how the questions are asked. In the present analysis, we used only a single item from the COPSOQ questionnaire which asks about the community at the workplace and not the department specifically, whereas the social capital within teams is a 9-item questionnaire. Thus, the social capital questions may be more robust and more directed to the work environment at the department which suggestively can be improved by performing workplace exercise together with colleagues. Moreover, social support from supervisors did not differ between the groups at follow-up. However, this is in line with the aforementioned social capital analysis which showed that social capital between the worker and management did not improve in response to workplace exercise [17]. Similarly, no effect was seen for job influence. This is, however, in contrast with the study by Chanchai and co-workers who demonstrated that 6 months of participatory ergonomics can improve job influence [40]. However, that study specifically encouraged the workers to adjust their work tasks, whereas the present study did not focus on altering the working conditions, but on incorporating small physical exercise sessions during the day with the aim of reducing musculoskeletal pain. Nevertheless, the present intervention may be of too short duration for altering the workers behavioral patterns.

We also included control questions that were not expected to change as results of the intervention. Working at a hospital may impose emotionally demanding situations, but are not expected to be influenced by physical exercise. As expected, emotional demands did not change following either of the exercise interventions as the type and amount of patients were somewhat constant through the intervention period.

Interestingly, a main effect was observed for work pace meaning that the workers who performed workplace exercise had to work faster more often compared with the home group. A logical explanation for this is that the total amount of tasks the department had to do did not change because they participated in the study, neither did the hospital hire additional staff to fill in the missing time from work due to training. Thus, as no additional help was provided during the intervention period the workers had to catch up, i.e. work faster, with their tasks when returning from the daily scheduled 10 minutes exercise session. One way to cope with this problem is to provide more flexibility by offering multiple exercise sessions per day or longer time slots for instruction so the workers, more easily, can plan when to do the exercises with as little interference with their workday as possible. Nevertheless, offering additional and longer sessions is costly for the workplace and the impact of such initiatives should be investigated in a cost-effectiveness analysis. On the other hand, if the workplace does not have any additional costs in terms of hiring additional staff, there is no real expense for the hospital in spite of some minutes being used for physical exercise. Furthermore, the workers only participated in 2.2 out of the 5 offered exercise sessions per week, i.e. approximately 20–30 min per person. Yet, they may have increased their work pace in an attempt to free up some time to participate in the sessions. Importantly, the change in work pace did not negatively affect i.e. the present change in vitality or pain intensity [22] and need for recovery after the working day [32]. However, long-term studies are needed to fully evaluate whether the benefits of exercise can counteract the impact of increased work pace.

A strength of the present study design is that both interventions were active and neither of the interventions aimed at improving psychosocial factors, per se, which therefore minimizes the bias of outcome expectations and the associated placebo effects [41, 42]. A limitation on the other hand is that several of the psychosocial questions used in the present analysis were single questions from a multiple item questionnaire which potentially may lead to less stable values than when an average of several items is used and therefore more conservative outcomes. Another limitation when interpreting the present results is the difference in training adherence between the groups. The workplace group participated in the training, on average, 2.2 times per week whereas the home group trained 1.0 times per week. Accordingly, the higher training adherence in the workplace group may potentially explain some of the improvements found in vitality and concern and control of pain. A potential method to increase training adherence in the home-based exercise group would be to send out encouraging text messages frequently. This procedure has recently shown to increase general physical activity along with accompanied reductions in fat mass and without compromising productivity among ambulatory clinical nursing staff [43]. However, the present study was designed as a pragmatic study that tested whether investing in working hours, instructors and coaches is more effective than training at home with simple equipment and without additional encouragement from the workplace. Thus, the present study investigated whether employee health can be maintained or enhanced with minimal investments and employer involvement or if larger investments are necessary.

## Conclusions

In conclusion, performing physical exercise together with colleagues during working hours is accompanied with higher training adherence and is more effective than home-based exercise in improving vitality and concern and control of pain among healthcare workers. Thus, group-based workplace interventions aiming at relieving pain may induce physiological as well as psychosocial benefits.
